# Melatonin and mesenchymal stem cells co-administration alleviates chronic obstructive pulmonary disease via modulation of angiogenesis at the vascular-alveolar unit

**DOI:** 10.1007/s00424-024-02968-3

**Published:** 2024-05-14

**Authors:** Mira Hanna, Sabreen Sayed Elnassag, Dina Hisham Mohamed, Marawan Abd Elbaset, Olfat Shaker, Effat A. Khowailed, Sarah Ali Abdelhameed Gouda

**Affiliations:** 1 Department of Medical Physiology, Faculty of Medicine, Kasr Al-Ainy, Cairo University, El-Maniel 11451, Cairo, Egypt; 2 Department of Histology, Faculty of Medicine, Cairo University, El-Maniel 11451, Cairo, Egypt; 3 Department of Pharmacology, Medical Research and Clinical Studies Institute, National Research Centre, Cairo, Egypt; 4 Department of Biochemistry, Faculty of Medicine, Kasr Al-Ainy, Cairo University, El-Maniel 11451, Cairo, Egypt

**Keywords:** COPD, Melatonin, MSCs, Angiogenesis, VEGF, HIF

## Abstract

Chronic obstructive pulmonary disease (COPD) is considered a severe disease mitigating lung physiological functions with high mortality outcomes, insufficient therapy, and pathophysiology pathways which is still not fully understood. Mesenchymal stem cells (MSCs) derived from bone marrow play an important role in improving the function of organs suffering inflammation, oxidative stress, and immune reaction. It might also play a role in regenerative medicine, but that is still questionable. Additionally, Melatonin with its known antioxidative and anti-inflammatory impact is attracting attention nowadays as a useful treatment. We hypothesized that Melatonin may augment the effect of MSCs at the level of angiogenesis in COPD. In our study, the COPD model was established using cigarette smoking and lipopolysaccharide. The COPD rats were divided into four groups: COPD group, Melatonin-treated group, MSC-treated group, and combined treated group (Melatonin–MSCs). We found that COPD was accompanied by deterioration of pulmonary function tests in response to expiratory parameter affection more than inspiratory ones. This was associated with increased Hypoxia inducible factor-1α expression and vascular endothelial growth factor level. Consequently, there was increased CD31 expression indicating increased angiogenesis with massive enlargement of airspaces and thinning of alveolar septa with decreased mean radial alveolar count, in addition to, inflammatory cell infiltration and disruption of the bronchiolar epithelial wall with loss of cilia and blood vessel wall thickening. These findings were improved significantly when Melatonin and bone marrow-derived MSCs were used as a combined treatment proving the hypothesized target that Melatonin might augment MSCs aiming at vascular changes.

**Supplementary Information:**

The online version contains supplementary material available at 10.1007/s00424-024-02968-3.

## Introduction

Chronic obstructive lung disease (COPD) is a common disease with high morbidity and mortality with no present effective treatment. It occurs due to widespread smoking, infection, and pollution. The main pathological features of COPD include bronchitis, irreversible damage of airways, and emphysematous changes [15, 60]. It is also identified by abnormality of pulmonary vascularization with increased microvascular permeability [46]. Forty genes have been linked to the signaling and regulation of cell apoptosis and angiogenesis [28]. However, the detailed mechanisms concerned in COPD pathogenesis remain vague.

Angiogenesis changes in COPD are a crucial complex multiphase process that includes a lot of growth factors, cytokines, and chemokines. It is essential in the airway remodeling step for irreversible airway obstruction. In addition, it may be the pass to the development of lung cancer as a complication of COPD [44, 59]. Vascular endothelial growth factor (VEGF), a potent multifunctional cytokine, is one of many proteins that potentially affect angiogenesis. In addition, it is widely expressed in highly vascularized organs, including the lung. Being crucial for endothelial cell maintenance and proliferation in asthma and COPD, VEGF absence leads to endothelial cell apoptosis. Nevertheless, its expression increased in chronic inflammation and fibrosis which might play a role in the pathogenesis of emphysema through apoptosis and oxidative stress pathways. It might have opposing functions according to the site of action, a damaging function in the bronchi and a protective one in the alveoli. The exact role of VEGF in the pathogenesis of different stages of COPD is still controversial [59].

VEGF is modulated by many factors including nitric oxide and fibroblast growth factor. Meanwhile, its expression is induced primarily by hypoxia-inducible factor-1α (HIF-1α) which is a transcription factor activated by hypoxia. It plays an important role in oxygen homeostasis in which it facilitates oxygen delivery and utilization by affecting vascular remodeling, angiogenesis, redox homeostasis, and glucose metabolism [27, 52, 64, 89].

Melatonin, a pineal gland hormone, plays an important role as an anti-inflammatory, antioxidant, and antiapoptotic agent. Therefore, it has been suggested that Melatonin could have a protective effect in a lot of pulmonary diseases such as acute lung injury, acute respiratory distress syndrome, sepsis-induced lung injury, and COPD [76, 83]. It has been reported that Melatonin may suppress angiogenesis through depressing the HIF-1α/VEGF pathway [17, 58].

Mesenchymal stem cells (MSCs) have a lot of potential to improve tissue repair. They could play a role in the treatment of many diseases due to their anti-inflammatory and immune regulatory impact. In addition, they reside in many tissues including the lungs. So, they may have a lung tissue repair impact through paracrine effect on damaged alveolar tissue and endothelial integrity. One of the growth factors secreted by MSCs is VEGF which may play a pivotal role in lung diseases [9, 25, 62]. A question introduces itself that in COPD, VEGF increases in most of the studies while MSCs improve COPD even though they secrete VEGF. So, it may be a pitfall that needs further investigation.

We hypothesize that using Melatonin could improve the internal environment for better action of bone marrow-derived MSCs by improving angiogenesis through adjusting VEGF. In this research experiment, we introduced the first step in our project aiming to detect the impact of co-administration of Melatonin and MSCs on the expression of HIF-1α/VEGF in relation to lung functions in COPD.

## Materials and methods

### Experimental animals

Fifty male Wistar rats with body weights ranging from 160 to 200 g were used in this study. Animals were purchased and kept in the Animal House of Kasr Al-Ainy, Faculty of Medicine, Cairo University. All rats were kept in chip-bedded cages at room temperature under a normal day-night cycle. All animals were kept under the same environmental conditions and given free access to food and water for the entire duration of the study. Experimental animal protocols and animal procedures complied with the highest International Criteria of Animal Experimentation and were approved by the Institutional Animal Care and Use Committee (IACUC), Cairo University (Approval number CU-III-F-50–21).

### Experiment design

The study lasted for 2 months, which started with 50 rats. Ten rats were randomly considered the control group, and the remaining 40 rats were exposed to cigarette smoking and lipopolysaccharides (LPS) administration to induce COPD. After inducing COPD, the rats were subdivided into further study groups. The following five main groups in this study are as follows: Group I, control group (*n* = 10), in which saline was administered instead of LPS; Group II, COPD group (*n* = 10); Group III, Melatonin-treated group (Melatonin) (*n* = 10); Group IV, MSC group bone marrow-derived mesenchymal stem cell-treated group (MSCs) (*n* = 10); Group V, combined treated group (Melatonin–MSCs) group (*n* = 10).

### Induction of COPD in adult male rats

COPD was induced as discussed by [71]. Briefly, the rats were exposed to cigarette smoke (CS) of 12 cigarettes for 4 weeks twice daily with 2-h free intervals in a Plexiglas tobacco smoke chamber. LPS (Biospes cat# BCS1084) was intratracheally (IT) injected on the 1st and 15th days of exposure to CS in a dose of 200 µg/200 µL/rat. During LPS IT instillation, the rats were anesthetized using ketamine and xylazine combination at doses of 80–100 mg/kg and 20 mg/kg IP, respectively. Then, they were put on a warming mattress in a 45° position during instillation to maintain body temperature at 37 ± 0.5 °C until the rats had recovered from anesthesia. After instillation, percussion of the rat chest in different positions was done to ensure distribution along the lungs.

### Isolation of bone marrow-derived MSCs (BM-MSCs)

MSCs were obtained from rat bone marrow by washing out the cells from femurs and tibias of 6-week-old male Wistar rats with Dulbecco’s modified Eagle’s medium (DMEM) (Thermo Fisher, cat NO 11960044) containing 1% penicillin/streptomycin. Then, 30-min centrifugation of cell suspension was performed. Followed by resuspension of the cells, pellet was in DMEM containing 10% fetal bovine serum and then plated at a density of 1 × 10^6^ cells/cm^2^ and cultured at 37 °C in a 5% CO_2_ incubator. Twenty-four hours later, non-adherent cells were washed from the cultured dishes with PBS, and a fresh medium was added to be changed every 3–4 days until confluency was detected. In identifying the BM-MSCs (supplementary Fig. 1 and 2), surface marker antigens were identified using flowcytometry. Plastic adherent MSCs expressed CD105 and CD90 but did not appear to express CD45 and CD34 [69, 70, 72]. Homing was also detected by labeling MSCs with PKH26. Labeling of stem cells with PKH26, which is a fluorescent dye called Paul Karl Horan-26 (Sigma Company, Egypt), was used to label MSCs that were obtained from the second passage to trace homing of stem cells in the lung. The cells were pelleted, washed in a medium devoid of serum, and then labeled. Approximately 4 × 10^6^ MSCs were delivered in 500 µL of PBS by intratracheal instillation after the induction of the disease model [87]. The labeled MSCs were then examined in unstained lung sections under a fluorescent microscope to visualize and track their presence (supplementary Fig. 3).

### Melatonin

Ten-milligram capsules were prepared as described previously by Hanna et al. [30] and were given with a dose of 30 mg/kg once daily for 30 days by oral gavage after the induction of the disease model [66].

### Non-invasive measurement of pulmonary function tests

Pulmonary function tests were performed at the end of the experimental protocol for all studied groups by using the PowerLab spirometry of (AD-Instruments spirometer, PowerLab/8SP, ML140) and head-out body plethysmography. The rats were placed in body plethysmographs with their heads protruding through the neck collar of a dental latex dam and into a head exposure chamber connected to a bridge amplifier to test lung functions [57]. When the rat readings reached a stable level (known as the steady state, which takes place after around 5 min for acclimatization), pulmonary function monitoring was initiated. The respiratory flow was measured as the flow through a calibrated pneumotachograph attached to the plethysmograph and produced by the thoracic movements of the rat. The flow was measured using a differential pressure transducer coupled to the pneumotachograph. The tidal volume (VT) of the spontaneously breathing rat in milliliter, and the time of inhalation and expiration (TI, TE; time taken to inspire/exhale) was obtained from the amplified flow signals, and the forced expiration, forced vital capacity (FVC), forced expiratory volume in 1 s (FEV1), FEV1/FVC ratio, peak inspiratory flow (PIF), and peak expiratory flow (PEF) were detected [33].

### Quantitative real-time PCR (qPCR) for detection of the HIF gene

Lung tissue was collected and homogenized for RNA extraction according to the manufacturing protocol. Total RNA was extracted using miRNeasy mini kit (Qiagen, Valencia, CA, USA). Quantitation and assessment of RNA purity assessment were done using the NanoDrop® (ND)-1000 spectrophotometer (NanoDrop Technologies, Inc. Wilmington, USA). cDNA was done in a final volume of 20 µL RT reactions using the RT kit (Qiagen, Valencia, CA, USA). Quantitative real-time PCR (qPCR) for detecting of the HIF gene was carried out using SYBR® Green PCR kit and protocol for RNA quantitative detection (Qiagen, Valencia, CA, USA).

### Assessment of VEGF

Lung tissue was weighed and then homogenized in 200 µl PBS. After centrifugation at 4000 × g, the supernatant was separated and used for determination of VEGF level by using an ELISA kit (Bioassay Technology Laboratory (Cat. No E2557Hu), Zhejiang, China) according to the manufacture protocol.

### Histological examination of lung tissue

#### Light microscopic examination

The right lung was isolated, and lung specimens were fixed intratracheally in 10% formol saline for 24–48 h, dehydrated in ascending grades of alcohol (70%, 95%, 100%), cleared in xylene, and then embedded in paraffin. Serial sections of 7-µm thickness were cut and subjected to Hematoxylin & Eosin (H&E) stain for histological evaluation [43].

#### Immunohistochemistry

CD31 staining purified monoclonal mouse anti-rat CD31 antibody (BD Pharmingen) was used to quantify the microvascular bed using avidin–biotin-peroxidase complex technique. CD31-positive cells showed a brown membranous reaction. Tonsil sections were used as positive control specimens, and one of the lung sections was used as a negative control skipping the step of applying the primary antibody [73].

#### Morphometry

Data were acquired using the “Leica Qwin 500 C” image analyzer computer system Ltd. (Cambridge, England) in the Medical Histology and Cell Biology Department, Faculty of Medicine, Cairo University. The image analyzer included a colored video camera (Olympus), colored monitor, and hard disc of an IBM personal computer linked to the microscope and processed by the “Leica Qwin 500 C” software. The image analyzer was first calibrated to automatically convert the measurement units (pixels) produced by the image analyzer program into actual micrometer units. Slides were examined under the light microscope, and the following parameters were measured:

- Radial alveolar count in H&E-stained slides: measured in 200 × H&E-stained fields by drawing a perpendicular line from the center of a respiratory bronchiole to the nearest definitive alveolar septal wall [56]
- Area % of + ve CD31 immunoreactivity

#### Statistical methods

Data were coded and entered using the statistical package for the Social Sciences (SPSS) version 28 (IBM Corp., Armonk, NY, USA). Before the statistical analysis, data values were checked for normality using the Shapiro test and homogeneity by Levene’s test. The data are presented as means ± SD. Comparisons between groups were made using Student’s *T* test or analysis of variance (ANOVA) with multiple comparison post hoc test in normally distributed quantitative variables. In contrast, the non-parametric Kruskal–Wallis test and Mann–Whitney test were used for non-normally distributed quantitative variables [11]. *P*-values less than 0.05 were considered statistically significant.

## Results

### *Co-administration of MSCs and Melatonin improved pulmonary function tests in the COPD Wistar rat model*

**Fig. 1:**
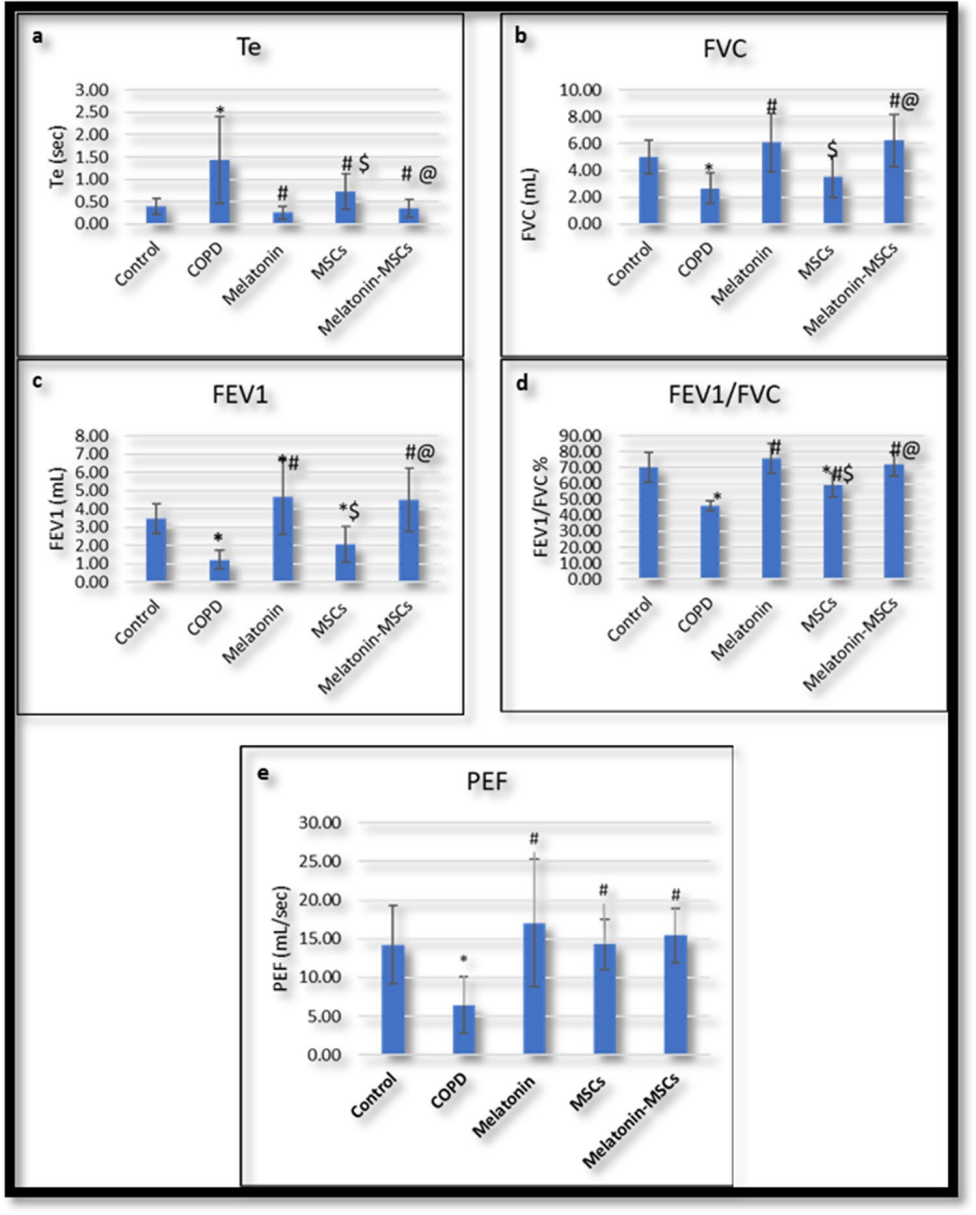
a–e The statistical results of the pulmonary function tests between different treated groups. *Statistically significant vs control group (*P* < 0.05). #Statistically significant vs COPD group (*P* < 0.05). $Statistically vs Melatonin group (*P* < 0.05). @Statistically significant vs MSC group (*P* < 0.05). Te, time expiratory; FVC, functional forced vital capacity; FEV1, forced expiratory volume one; PEF, peak expiratory flow

Induction of COPD affected most of the measured pulmonary function tests in all challenged rats. Relative to the control group, Te was significantly increased while FVC, FEV1, FEV1/FVC ratio, and PEF were significantly decreased in the COPD group (Fig. 1a–e). Interestingly, there was no significant difference in Ti, TV, and PIF in the COPD group versus in the control group (Table 1 in supplementary data). Oral Melatonin for 4 weeks significantly lowered the Te and significantly improved FVC, FEV1, FEV1/FVC ratio, and PEF compared to the COPD group. Apart from FEV1, there was no significant difference in these parameters compared to the control group (Table 2 in supplementary data). The BM-MSC single-treated group significantly decreased Te relative to the COPD group and increased the FEV1/FVC ratio and PEF compared to the COPD group, but regarding FVC and FEV1, there was no significant improvement in the MSC group relative to the COPD group. Additionally, MSCs could not normalize the affected parameters as there was a significant difference in FEV1 and FEV1/FVC ratio in the MSC group compared to the control group, although Te, FVC, and PEF showed no significant difference in the MSC group compared to the control group (Table 3 in supplementary data). Regarding Te, FVC, FEV1, FEV1/FVC ratio, and PEF, the Melatonin group showed significant improvement compared to the BM-MSC group. On the other hand, the combined treated group significantly lowered the Te and significantly improved the effect on FVC, FEV1, FEV1/FVC ratio, and PEF compared to the COPD group. Compared to the control group, values for Te, FVC, FEV1, FEV1/FVC, and PEF showed no significant difference noticed (Table 4 in supplementary data). In addition, the combined treatment significantly improved Te, FVC, FEV1, and FEV1/FVC ratio compared to the MSC group. It also showed no significant difference in Te, FVC, FEV1, FEV1/FVC, and PEF compared to the Melatonin group (Table 5 in supplementary data).

### MSCs and Melatonin co-administration improved the lung tissue VEGF level

**Fig. 2:**
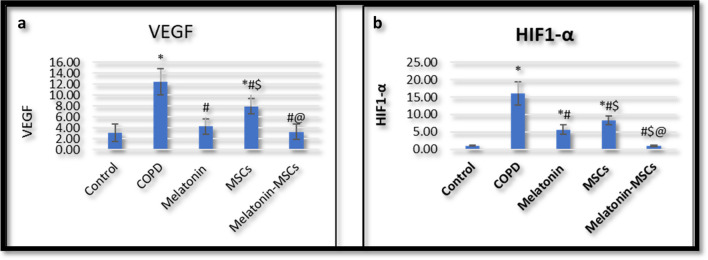
**a**, **b** The statistical results of VEGF and HIF between different treated groups. **a** Comparison of VEGF among the studied groups. **b** Comparison of HIF among the studied groups. *Statistically significant vs control group (*P* < 0.05). #Statistically significant vs COPD group (*P* < 0.05). $Statistically vs Melatonin group (*P* < 0.05). @Statistically significant vs MSC group (*P* < 0.05). VEGF, vascular endothelial growth factor; HIF-1α, Hypoxia inducible factor 1 alfa

By induction of COPD, there was a significant increase in VEGF expression level in lung tissue (Fig. 2a) as compared with the control group (Table 6 in supplementary data). The Melatonin-treated group showed a significant decrease in VEGF in lung tissue as compared with the COPD group, and there was no significant difference compared to the control group (Table 7 in supplementary data). The MSC-treated group showed a significant decrease in VEGF, as compared with the COPD group, while relative to the control group, there was still a significant difference (Table 8 in supplementary data). The combined treated group significantly decreased VEGF in lung tissue compared to the COPD group and no significant difference relative to the control group (Table 9 in supplementary data). Melatonin significantly improved VEGF expression compared to the MSC group. In addition, it showed that combined treatment had a significantly improving effect on VEGF relative to the MSC group. There was no significant improvement in VEGF in the combined group versus the Melatonin group (Table 10 in supplementary data).

### MSCs and Melatonin improved the expression of HIF-1α

The expression of HIF-1a in lung tissues of the COPD group was significantly increased (Fig. 2b) as compared to the corresponding values in control rats (Table 6 in supplementary data). The Melatonin-treated group showed a significant decrease in HIF-1α expression in lung tissue as compared with the COPD group. Additionally, compared to the control group, HIF-1α expression showed a significant difference in the Melatonin group (Table 7 in supplementary data). The MSC-treated group showed a significant decrease in HIF-1α expression as compared with the COPD group. At the same time, there was still a significant difference in the MSC group relative to the control group (Table 8 in supplementary data). The combined treated group significantly decreased HIF-1α expression in lung tissue compared to the COPD group, while HIF-1α showed no significant difference in the combined treated group relative to the control group (Table 9 in supplementary data). Melatonin significantly improved HIF-1α expression compared to the MSC group. On the other hand, the combined treated group showed a significant improvement in HIF-1α compared to the Melatonin group (Table 10 in supplementary group).

### Co-administration of MSCs and Melatonin alleviated histopathological changes of lung tissue

**Fig. 3:**
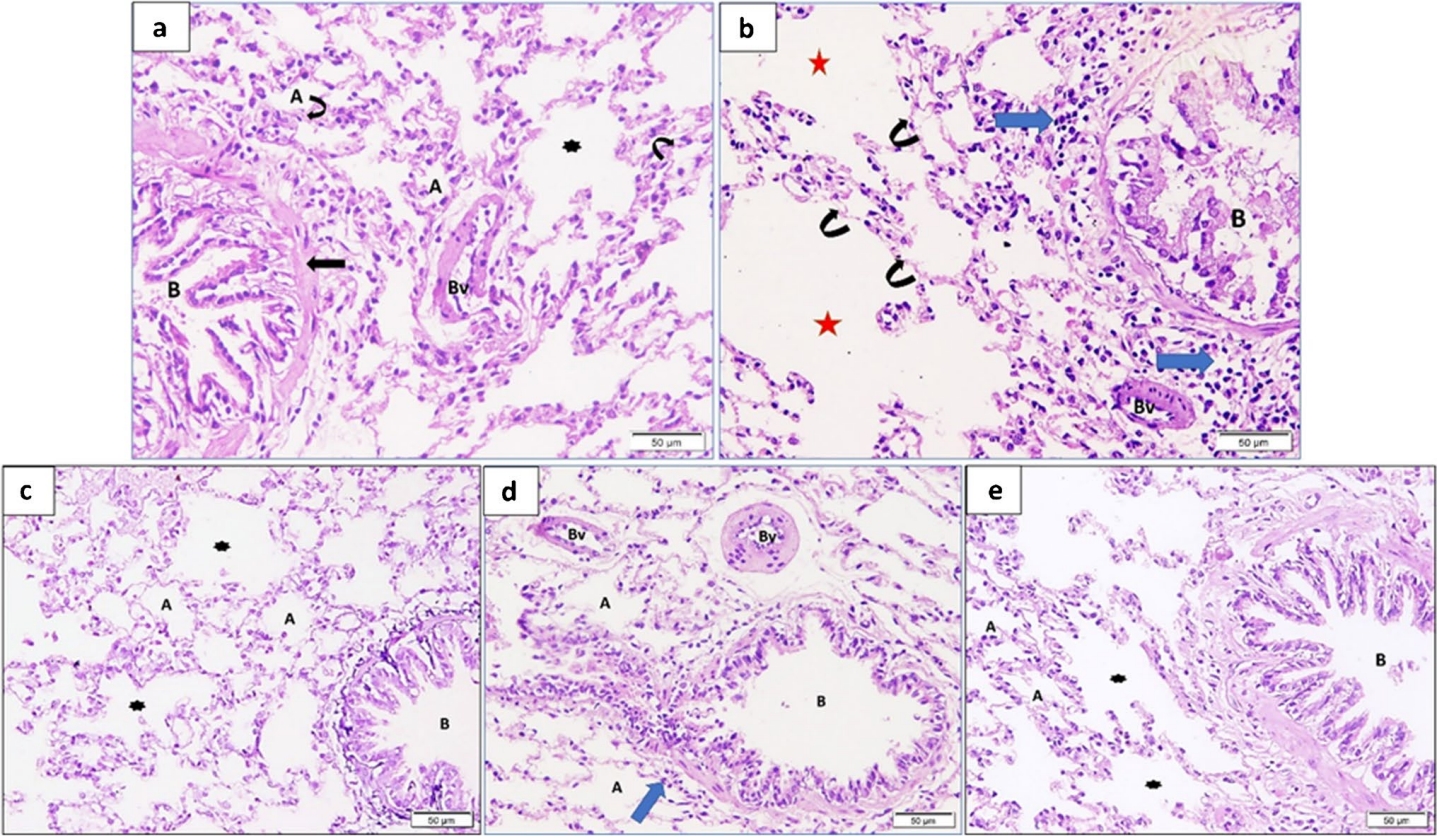
**a**–**e** Photomicrograph of lung section of the control group (**a**) showed air-filled spaces (alveoli) (A) separated by inter-alveolar septa (arrowheads) and alveolar sac (asterisk) and a bronchiole (B) surrounded by circularly arranged smooth muscles (arrow). Note the presence of a blood vessel (BV). The COPD group (**b**) showed massive enlargement of airspaces (red stars) and thinning of alveolar septa (curved arrows), in addition to, inflammatory cell infiltration (blue arrows) and disruption of the bronchiolar epithelial wall (B) with loss of cilia and blood vessel wall thickening (BV). The MSC-treated group (**c**) showed alveoli (A), some thickened blood vessels (Bv), and bronchiole (B) surrounded by some inflammatory cells (blue arrows) with loss of some of its cilia. The Melatonin-treated group (**d**) showed improved histological architecture. Alveoli (A), alveolar sacs (asterisk), and a bronchiole (B) were seen. The combined group (**e**) showed almost normal histological architecture with normally appearing alveoli (A), bronchiole (B), and some alveolar sacs (asterisk)

The COPD-induced group showed lung tissue destruction in the form of massive enlargement of airspaces and thinning of alveolar septa. In addition, there was inflammatory cell infiltration and disruption of the bronchiolar epithelial wall with loss of cilia with blood vessel wall thickening. The MSC-treated group showed relatively well-formed alveoli, some thickened blood vessels, and bronchioles surrounded by some inflammatory cells with loss of some of their cilia. The Melatonin-treated group showed improved histological architecture in which well-formed alveoli, alveolar sacs, and bronchioles were seen. The combined treated group showed almost normal histological architecture with normally appearing alveoli, bronchiole, and some alveolar sacs (Fig. 3a–e).

There was a significant decrease in the mean radial alveolar count in the COPD group as compared to the control group. The Melatonin-treated group showed a significant increase compared to the MSC group. The combined treated group showed a significant increase in this value compared to single-treated therapy by either melatonin or MSCs. Both the MSC-treated group and the Melatonin-treated group showed a significant decrease in radial alveolar count compared to the control group, while the combined treated group showed no significant difference in this value compared to the control group (Table 11 and Fig. 5a).

### Co-administration of MSCs and Melatonin improved the vascularity of lung tissue by adjusting the expression of CD31

**Fig. 4:**
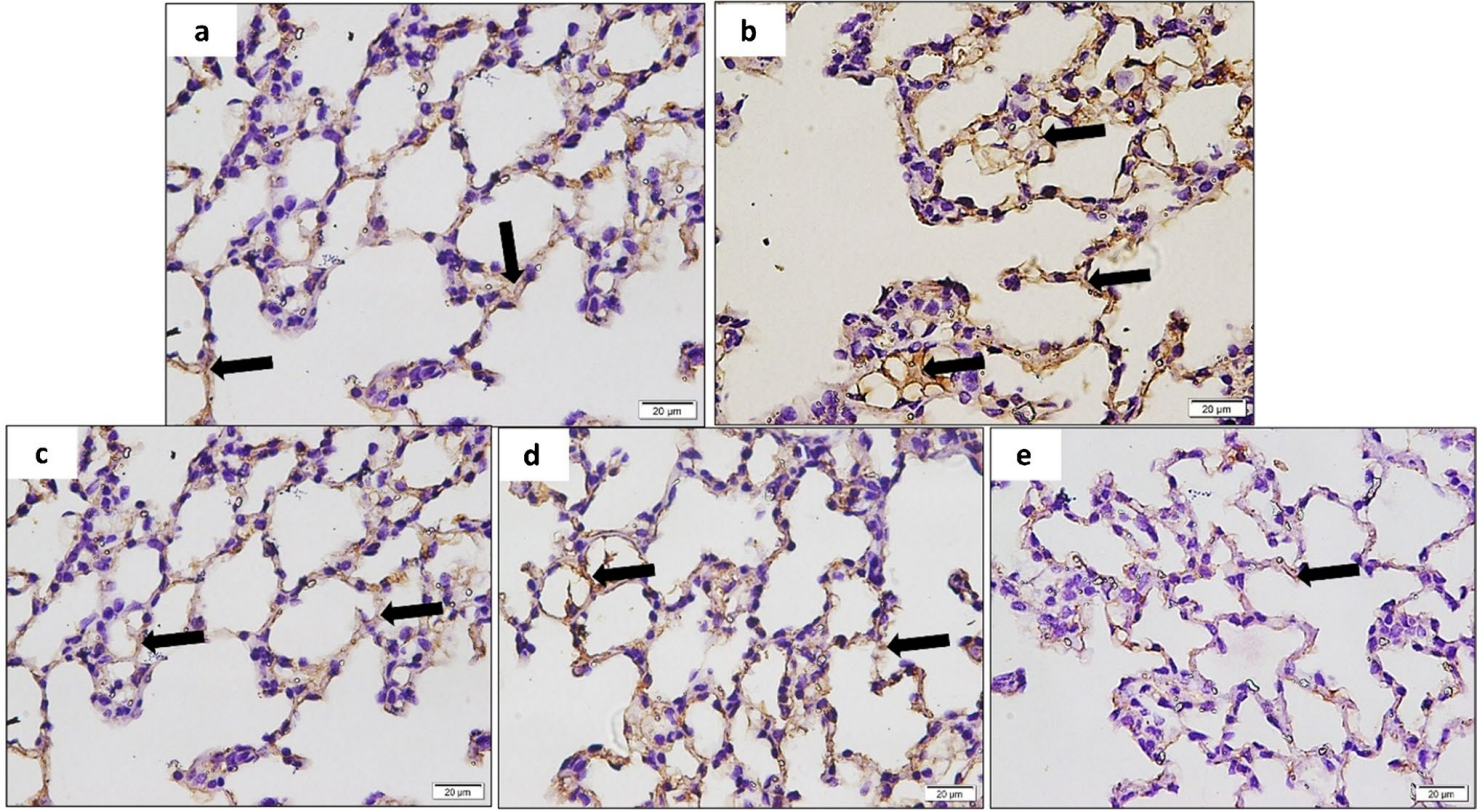
**a**–**e** A photomicrograph of the lung section of **a** the control group that showed positive CD31 immunoreactivity of endothelial cells of some alveoli, **b** the diseased group (COPD) that showed increased positive CD31 immunoreactivity of endothelial cells, **c** the MSC-treated group that showed moderate CD31 immunoreactivity, **d** the Melatonin-treated group that showed mild CD31 immunoreactivity, and **e** the combined treated (Melatonin and MSC-treated) group that showed minimal CD31 immunoreactivity

**Fig. 5:**
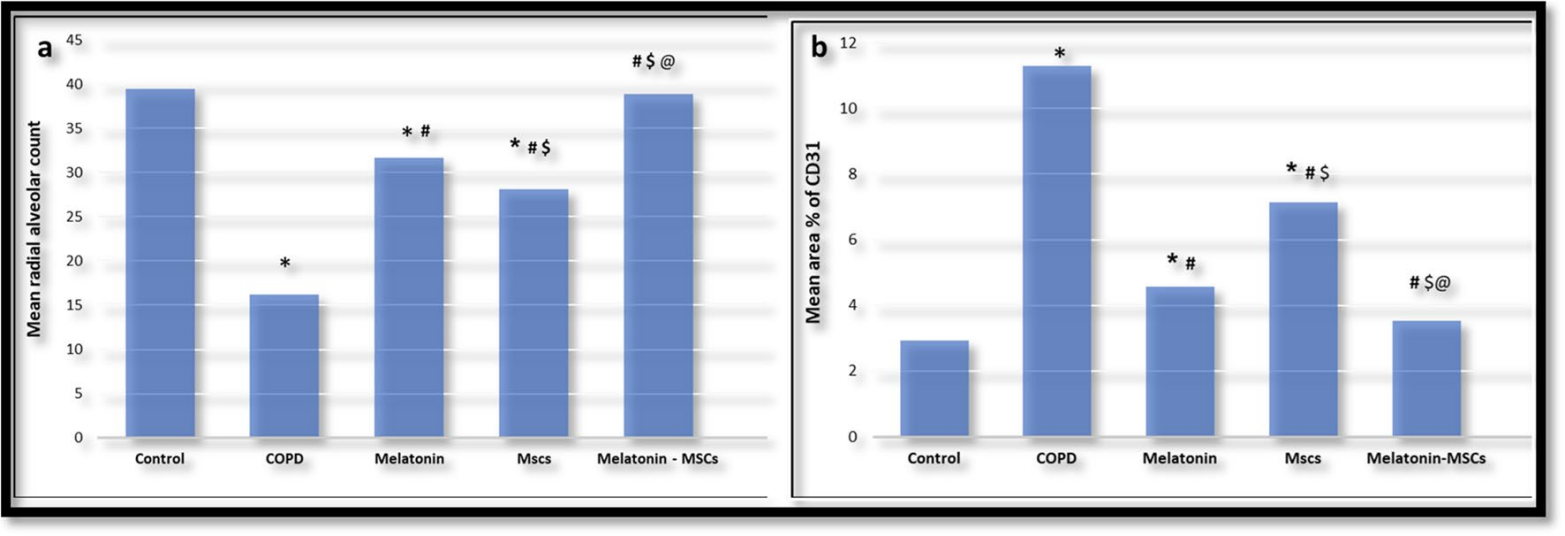
**a**, **b** The statistical comparison of mean radial alveolar count (**a**) and mean area % of CD31 (**b**) among the studied groups. Values are presented as mean ± SD. *Statistically significant vs control group (*P* < 0.05). #Statistically significant vs COPD group (*P* < 0.05). $Statistically vs Melatonin group (*P* < 0.05). @Statistically significant vs MSC group (*P* < 0.05)

The quantitative morphometric analysis of the mean area percent of positive CD31 immunoreactivity in CD31 stained lung sections showed that there was a significant increase in the mean area % of CD31 immunoreactivity in the COPD group as compared to the control group. The Melatonin-treated group showed a significant decrease in the mean area % of CD31 immunoreactivity compared to the MSC group. The combined treated group showed a significant decrease in the mean area % of CD31 immunoreactivity compared to single-treated therapy by either Melatonin or MSCs. Both the MSC-treated group and the Melatonin-treated group showed a significant increase in mean area % of CD31 immunoreactivity compared to the control group. In comparison, the combined treated group showed no significant difference in mean area % of CD31 immuno-reactivity compared to the control group (Figs. 4a–e and 5b; Table 12 in supplementary data).

## Discussion

In our study, we found that COPD was accompanied by deterioration of pulmonary function tests in response to expiratory parameter affection more than inspiratory ones. This was detected by the significant decrease in FEV1 and the prolonged time of expiration together with decreased FEV1/FVC ratio. This could be attributed to the fact that COPD is an obstructive lung disease that is usually accompanied by increased resistance to expiratory airflow that might have elongated the time of expiration and decreased volume of air expired during the first second. This could be due to airway narrowing or obliteration caused by mucus overproduction and chronic bronchial inflammation. In addition, accompanied emphysematous changes due to parenchymal destruction with reduced ability for gas exchange and refractory asthma might have a share in the expiratory functional deterioration [10, 47]. Previous studies were also in support of our study indicating airflow limitation mainly during the expiration demonstrated the same results showing that the expiratory lung function indices were affected in the COPD group [19, 55, 79, 82]. However, Xiao et al. [82] showed significant changes in inspiratory parameters as well, but they used different methods of induction and measurement techniques so as they induced COPD using cigarette smoking only for 28 weeks and stated that the inspiratory changes occurred after 14 weeks. Accordingly, we assumed that inspiratory functions would be affected more after prolonged exposure to cigarette smoking that may be due to exaggerated emphysematous changes.

Our results regarding HIF-1α and VEGF in lung tissues showed a significant increase in the COPD group compared to the control group [12, 18, 20]. Hypoxia, through HIF-1α, is the inducible factor that increases VEGF trying to maintain endothelial cells and alveolar epithelium and scavenging against apoptosis and severe emphysematous changes. Still, the persistence of the irritating factor which is smoking leads to overexpression of VEGF due to inflammation. On the other hand, one of the suggested causes of emphysematous changes is the decreased VEGF or its receptor blockade leading to apoptosis. It is suggested that the survival of lung endothelial cells depends chiefly on VEGF, so their apoptosis leads to the loss of capillaries that may consequently lead to emphysema [20, 48]. However, it was reported that VEGF levels were raised in the airways of both asymptomatic and COPD smokers. Moreover, there was a close correlation observed between VEGF levels in the airways and markers of airway inflammation [59]. In addition, there was a correlation in patients suffering from asthma who showed elevated levels of VEGF-A in bronchial biopsies, induced sputum, and bronchoalveolar lavage fluid with increased total airway vascular area and smaller airway caliber [5, 59]. The key between the increased VEGF level in case of COPD in addition to emphysematous change may be answered by further investigation of the types of its receptors’ expression and contribution to the pathway of the diseases.

The increased HIF-1α in our study could be attributed to that tissue hypoxia resulted from chronic inflammation. Consequently, tissue remodeling occurred because of declined O_2_ diffusion through thickened mucus, edematous tissues, and airways or through vascular shunting and diminished O_2_ delivery to the epithelium. Additionally, increased oxygen demand because of excessive O_2_ consumption by the epithelial cells leads to activation of the HIF-1α pathway. It was also stated that the activity of HIF-1 could be stimulated by hypoxia through the changes in the expression of HIF-1α mRNA and protein [18, 64].

Lung tissue of the COPD rats in the present study showed massive enlargement of the airspaces, thinning of alveolar septa, inflammatory cell infiltration, and disruption of the bronchiolar epithelial wall with loss of cilia and blood vessel wall thickening in addition to decreased radial alveolar count [50, 82]. Besides, there was a significant increase in positive CD31 immunoreactivity compared to the control group. Previous studies reported that CD31, the marker of angiogenesis, was released from pulmonary microvascular endothelial cells mainly in response to apoptosis induced by cigarette smoke [40, 74, 78]. The changes in the structure of alveolo-vascular unit that accompany COPD might be underlying the basic pathology as ventilation/perfusion ratio would have been distorted affecting lung functions. In addition, bronchial inflammation and hypertrophy together with vascular remodeling and angiogenesis shared the pathology [10, 22, 29, 80]. Kato et al. [38] demonstrated that increased expression of the genes related to vascular endothelial cells in blood cells from smokers was associated with the development of COPD. Also, CD 31 expression fluctuated throughout the disease course in which it decreased in moderate COPD and increased in severe COPD patients. They showed that its expression increased generally in smokers.

Melatonin and MSCs together acquired more impact on improving pulmonary function in COPD in the present study. Our results showed significant improvement in the pulmonary function parameters compared to the COPD group. Even more, there was no significant difference between these parameters in the Melatonin group compared to the control group indicating nearly adequate recovery. Melatonin administration has a better effect than MSC injection on expiratory parameters of the pulmonary function tests in COPD rats. Some studies showed the protective role of Melatonin in COPD tackling anti-inflammatory mechanisms. This could be related to that COPD was associated with inflammatory response, excessive accumulation of ROS, and abnormal excessive angiogenesis. Therefore, Melatonin improved the disease through its effect as potent antioxidant, anti-inflammatory, and anti-angiogenic [31, 66, 85]. Also, it has been investigated that in COPD patients with acute exacerbation, there was decreased serum Melatonin level with positive correlations with the deterioration of expiratory parameters which suggested the protective role of Melatonin [51].

In the present study, intratracheal injection of MSCs in COPD rats showed a significant improvement in expiratory parameters relative to the non-treated COPD group. However, this improvement showed a significant difference compared to the control group which indicates that in the internal environmental conditions of COPD, MSCs could not completely revert pulmonary functions.

The reported improvement in our results might be related to that using MSC treatment in COPD could contribute to tissue maintenance, regeneration, and modulation of immune responses through their known paracrine effect, induction of the release of anti-inflammatory molecules as showed by some studies [6, 35, 42, 54, 81]. In addition, it suppresses the production of pro-inflammatory mediators such as TNF-α, IL-1β, IL-6, and monocyte chemotactic peptide-1 and down-regulate cyclooxygenase-2 [3, 24, 36]. Through their exosomes’ secretion, MSCs promote also macrophage polarization from M1 macrophages with pro-inflammatory activity toward M2b macrophages with more phagocytic and anti-inflammatory activity which are essential for the resolution of inflammation and regenerative procedures [4], while the pulmonary function could not reach the control value because it was reported that oxidative stress could induce premature senescence of MSCs [93].

Most of the studies related to lung diseases checked the effect of MSC administration from histological and biochemical assessment without checking the pulmonary function effect [21, 24, 34, 63, 90, 91]. In addition, there are a lot of factors controlling the therapeutic effect of MSCs such as the source of MSCs, administration route, dosage, dose intervals, and the stage of the disease [14, 42, 72]. In a study done by Karaoz et al. [37], they illustrated that MSCs alleviated the severity of symptoms in patients suffering from COPD and markedly improved the pulmonary function parameters using four doses of MSCs in which FEV1/FVC ratios raised to normal levels. It also should be noted that high doses of MSCs might be tumorigenic as reported by Chen research group [13] that MSCs have strong proliferative properties; therefore, they recommended a strictly controlled number of MSCs for treatment of COPD [49]. Moreover, the results of clinical trials of MSC injection in COPD patients also support the idea that MSCs alone may be insufficient or may lead to transient improvement and failure of engagements [14, 26, 32, 72].

In the present study, the combined treated group showed significant improvement in expiratory pulmonary function parameters compared to the COPD group and showed no significant difference compared to the control group indicating adequate recovery. It was reported that preconditioning of MSCs with Melatonin could powerfully serve as an antioxidant and guard MSCs from oxidation injury by biologically eradicating free radicals and that in turn could protect the injected MSCs against premature senescence or early apoptosis after transplantation and inflate their therapeutic role in diseased tissues [61]. Despite that combined treatment significantly improved expiratory pulmonary function tests compared to the MSC group, there was no significant difference between it and the Melatonin group. So, it was suggested that the co-treatment of Melatonin and MSCs may have a better effect by improving the internal environment to which MSCs were subjected. Also, we assumed that Melatonin and MSCs might need longer duration for better effect on the physiological level or Melatonin should have been administrated earlier to augment the role of MSCs. Shigematsu et al. [65] reported in their study that proper improvement in expiratory parameters of pulmonary functions occurred after 2 months of MSC injection.

In addition, Melatonin in the present study showed a significant decrease in VEGF and HIF-1α expression in lung tissue compared to the COPD group. Inhibition of HIF-1α and VEGF, whether at the transcriptional level or HIF-1α degradation, is the main target of Melatonin for inhibition of angiogenesis and oxidative stress especially under hypoxic conditions [45, 68, 75, 86, 88]. Other studies showed that depending on the surrounding microenvironment, Melatonin either stimulates or inhibits neovascularization by various mechanisms, producing different biological effects. In gastric ulcers and skin lesions, Melatonin prevented the lesions by encouraging angiogenesis through upregulation of angiogenetic inducers [8], while Melatonin treatment was efficient in inhibiting angiogenesis through destructing the development of vessels inside the tumor tissue [39] by direct and indirect effects. As through the direct effect, Melatonin prevents the function of VEGF, while it indirectly hinders other growth factors and may undermine HIF-1α through its antioxidant activity [23].

Moreover, in this study, injected MSCs showed a significant decrease in VEGF level and HIF-1α expression in lung tissue compared to the COPD group but not accessing control values. MSCs could reduce HIF-1α expression in hypoxic tissue through increasing the activity of antioxidant enzymes and decreasing ROS accumulation [7]. So, this in turn could decrease VEGF expression. However, it was reported that injection of MSCs could accelerate angiogenesis by secreting numbers of growth factors such as VEGF, platelet-derived growth factor (PDGF), fibroblast growth factor (FGF), and transforming growth factor-β (TGF-β) [2]. Double contradictory effects of MSCs on VEGF have been reported. They found that MSCs could inhibit the inflammatory response and oxidative stress through the inhibition of HIF-1α and VEGF, but on the other hand, MSCs could promote angiogenesis and increase VEGF expression. So, MSCs slightly inhibit VEGF expression [2, 41]. In the current study, the Melatonin group showed a significant lowering effect on the expression of VEGF and HIF-1α in lung tissues relative to the MSC group.

The combined treated group showed a significant decrease in VEGF and HIF-1α expression relative to the COPD group. The co-treatment with Melatonin might have balanced that angiogenic effect. In accordance with our results, it was reported that injection of MSCs preconditioned by Melatonin in rats resulted in a significant decrease in the levels of HIF-1α mRNA and VEGF expression [1]. In contrast to our results, Zheng and his colleagues [92] demonstrated that bone marrow-derived MSCs treated with Melatonin in vitro and treated ovariectomized rats with osteoporosis in vivo showed higher expression levels of osteogenesis- and angiogenesis-related markers including VEGF compared to the untreated group. However, the high-dose-treated group was more effective than the low-dose treated suggesting that Melatonin therapy may display a dose-dependent manner especially in vivo.

Although our combined treated group showed improvement in the level of VEGF and HIF-1α expression compared to the Melatonin group, the improvement of VEGF level was not significant. This might be attributed to the mechanism used to assess HIF-1α that was PCR genetic expression assessment while that of VEGF was protein level assessment by ELIZA in which the last method may need more time to reach a significant level. Also, the dose-dependent impact of MSCs might have played a role in the discrepancy of the results of HIF and VEGF. Moreover, Melatonin is known to inhibit the pathway of cell senescence and preserve the expression of genes governing stemness as it is a potent antioxidant and anti-angiogenic factor. So, Melatonin could limit the pro-angiogenic effect of MSCs. Thus, Melatonin not only prepared a better environment for MSC action, but they both acted synergistically as anti-inflammatory and antioxidant agents resulting in a better improving effect [67].

In this study, consequently, to the previous biochemical results, the co-treatment of Melatonin and MSCs showed almost normal histological architecture with normally appearing alveoli and bronchioles, mean radial alveolar count, and a significant decrease in the mean area percent of CD31 compared to both Melatonin and MSC groups. Depending on the previously discussed role of Melatonin [51, 84], the Melatonin-treated group showed marked improvement in the histological architecture including alveoli and bronchioles in addition to a significant decrease in the mean area percent of CD31 [77] relative to the COPD group and the MSC group. While the combined treated group showed significant improvement compared to the Melatonin group, the MSC-treated group revealed that there was improvement [90]. However, the alveoli and bronchioles were still surrounded by some inflammatory cells and thickened blood vessels with minimal decrease in the mean area percent of CD31, and these changes indicated the effect of microenvironmental conditions on hindering MSC role. MSC supplementation could enhance the CD31 expression due to its pro-angiogenic effect [16, 53]. However, the dysfunction of injected MSCs under disease conditions was reported in relation to oxidative stress, thermal injury, and hypoxia [61].

The better improvement was detected in the Melatonin-treated group compared to the MSC-treated group; in addition to that, the Melatonin group showed less significant improvement when compared to the combined treated group. This supports our hypothesis that Melatonin administration together with MSCs might have a better effect on lung tissue in the COPD model and enhance the MSC effect. Along the same line, it has been reported that Melatonin administration could efficiently preserve self-renewal and differentiation properties of MSCs in culture dish after long-term passaging, and Melatonin allowed better regenerative function of MSCs through improving the surrounding microenvironment [67].

## Conclusion

COPD is a severe disease that needs more studies of pathophysiological pathways to reach a definitive therapeutic tool. Co-administration of Melatonin together with bone marrow-derived MSCs improved the outcome of expiratory pulmonary functions through improving angiogenesis at the level of vascular-alveolar unit by adjusting VEGF level together with HIF-1α expression.

### Supplementary Information

Below is the link to the electronic supplementary material.

Supplementary file1 (DOCX 11691 KB)

## Data Availability

All data generated or analyzed during this study are included in this published article and its supplementary information files.

## Abbreviations

COPD: Chronic obstructive lung disease
FEV1: Forced expiratory volume 1
FVC: Forced vital capacity
HIF-1α: Hypoxia-inducible factor-1α
LPS: Lipopolysaccharide
MSCs: Mesenchymal stem cells
PEF: Peak expiratory flow
PIF: Peak inspiratory flow
Te: Expiratory time
Ti: Inspiratory time
VEGF: Vascular endothelial growth factor
VT: Tidal volume
