# Supplementary material for: Melatonin and mesenchymal stem cells co-administration alleviates chronic obstructive pulmonary disease via modulation of angiogenesis at the vascular-alveolar unit
Source: Pflugers Arch. 2024 May 14;476(7):1155–68. doi: 10.1007/s00424-024-02968-3 (PMC11166745; doi:10.1007/s00424-024-02968-3)
Supplement: Supplementary file 1 — Supplementary file1 (DOCX 11691 KB) [file 424_2024_2968_MOESM1_ESM.docx]

Supplementary data:

Figures:


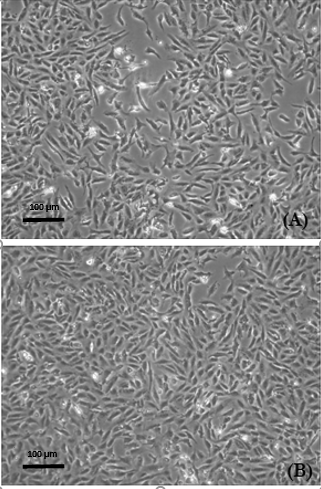


**Figure 1:** shows bone marrow derived Mesenchymal stem cells culture morphology adherent to the plastic culture dish 10X.


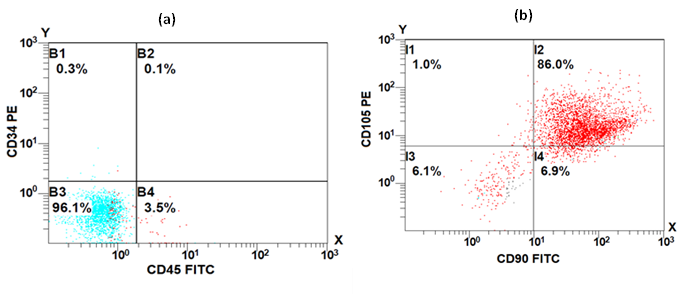


**Figure 2:** Flow cytometric characterization analyses of MSCs shows (a) negatively stained cells for CD45 and CD 34 and (b) positively stained cells for CD 90 and CD 105.

**Figure 3:** Photomicrograph of lung unstained section related to MSCs group **(a)** and combined MSCs and Melatonin group **(b)** demonstrating green colored fluorescence of homed MSCs labelled with PKH-26 in lung tissue (PKH26 fluorescent dye, (x 200).

|  | **Control** | **COPD** | **% change** |
| --- | --- | --- | --- |
| **TV (ml)** | 0.81±0.39 | 0.63±0.23 | -22.22 |
| **Ti (sec)** | 0.25±0.2 | 0.29±0.12 | 16.00 |
| **Te (sec)** | 0.4±0.18 | 1.44±0.97 * | 260.00 |
| **FVC (ml)** | 4.99±1.26 | 2.68±1.14 * | -46.29 |
| **FEV1 (ml)** | 3.47±0.82 | 1.22±0.51 * | -64.84 |
| **FEV1/FVC (%)** | 70.47±9.27 | 46.16±3.27 * | -34.50 |
| **PIF (ml/min)** | 3.22±1.54 | 2.15±0.99 | -33.23 |
| **PEF (ml/min)** | 14.24±5.04 | 6.46±3.65 * | -54.63 |

**Table1:** Effect of induction of COPD on pulmonary functions in adult male rats**.** Values are presented as mean ±SD. *: statistically significant compared to corresponding value in control group (P<0.05)

|  | **Control** | **COPD** | **Melatonin** | **% change compared to control** | **% change compared to COPD** |
| --- | --- | --- | --- | --- | --- |
| **TV (ml)** | 0.81±0.39 | 0.63±0.23 | 0.94±0.34# | 16.05 | 49.21 |
| **Ti (sec)** | 0.25±0.2 | 0.29±0.12 | 0.17±0.09 | -32.00 | -41.38 |
| **Te (sec)** | 0.4±0.18 | 1.44±0.97 * | 0.26±0.14 # | -35.00 | -81.94 |
| **FVC (ml)** | 4.99±1.26 | 2.68±1.14 * | 6.07±2.19 # | 21.64 | 126.49 |
| **FEV1 (ml)** | 3.47±0.82 | 1.22±0.51 * | 4.68±2.05 *# | 34.87 | 283.61 |
| **FEV1/FVC**  **(%)** | 70.47±9.27 | 46.16±3.27 * | 75.69±9.34 # | 7.41 | 63.97 |
| **PIF (ml/min)** | 3.22±1.54 | 2.15±0.99 | 2.70±0.91 | - 16.15 | 25.58 |
| **PEF**  **(ml/min)** | 14.24±5.04 | 6.46±3.65 * | 17.04±8.23# | 19.66 | 163.78 |

**Table2:** Effect of Melatonin on pulmonary functions in adult male rats. Values are presented as mean ±SD. *: statistically significant compared to corresponding value in control group (P<0.05), #: statistically significant compared to corresponding value in COPD group (P<0.05).

|  | **Control** | **COPD** | **MSCs** | **% change compared to control** | **% change compared to COPD** |
| --- | --- | --- | --- | --- | --- |
| **TV (ml)** | 0.81±0.39 | 0.63±0.23 | 0.59±0.26 | -27.16 | -6.34 |
| **Ti (sec)** | 0.25±0.2 | 0.29±0.12 | 0.23±0.12 | -8.00 | -20.69 |
| **Te (sec)** | 0.4±0.18 | 1.44±0.97 * | 0.72±0.40 # | 80 | -50 |
| **FVC (ml)** | 4.99±1.26 | 2.68±1.14 * | 3.53±1.56 | -29.26 | 31.72 |
| **FEV1 (ml)** | 3.47±0.82 | 1.22±0.51 * | 2.09±0.96 * | -39.77 | 71.31 |
| **FEV1/FVC**  **(%)** | 70.47±9.27 | 46.16±3.27* | 58.91±7.5 *# | -16.40 | 27.62 |
| **PIF (ml/min)** | 3.22±1.54 | 2.15±0.99 | 3.30±1.43 | 2.48 | 53.49 |
| **PEF (ml/min)** | 14.24±5.04 | 6.46±3.65* | 14.31±3.27 # | 0.63 | 121.52 |

**Table 3:** Effect of MSCs on pulmonary functions in adult male rats. Values are presented as mean ±SD. *: statistically significant compared to corresponding value in control group (P<0.05) #: statistically significant compared to corresponding value in COPD group (P<0.05)

|  | **Control** | **COPD** | **Melatonin -**  **MSCs** | **% change compared to control** | **% change compared to COPD** |
| --- | --- | --- | --- | --- | --- |
| **TV (ml)** | 0.81±0.39 | 0.63±0.23 | 1.05±0.22 # | 29.63 | 66.67 |
| **Ti (sec)** | 0.25±0.2 | 0.29±0.12 | 0.25±0.24 | 0.00 | -13.79 |
| **Te (sec)** | 0.4±0.18 | 1.44±0.97 * | 0.35±0.2 # | -12.50 | -75.69 |
| **FVC (ml)** | 4.99±1.26 | 2.68±1.14* | 6.23±1.99 # | 24.85 | 132.46 |
| **FEV1 (ml)** | 3.47±0.82 | 1.22±0.51 * | 4.49±1.72 # | 29.39 | 268.03 |
| **FEV1/FVC (%)** | 70.47±9.27 | 46.16±3.27 * | 72.28±7.52 # | 2.57 | 56.59 |
| **PIF (ml/min)** | 3.22±1.54 | 2.15±0.99 | 3.25±1.54 | 0.93 | 51.16 |
| **PEF**  **(ml/min)** | 14.24±5.04 | 6.46±3.65 * | 15.42±3.46 # | 8.29 | 138.70 |

**Table 4:** Effect of Melatonin & MSCs on pulmonary functions in adult male rats Values are presented as mean ±SD. *: statistically significant compared to corresponding value in control group (P<0.05), #: statistically significant compared to corresponding value in COPD group (P<0.05).

|  | **Control** | **COPD** | **Melatonin** | **MSCs** | **Melatonin - MSCs** |
| --- | --- | --- | --- | --- | --- |
| **TV(ml)** | 0.81±0.39 | 0.63±0.23 | 0.94±0.34# | 0.59±0.26 $ | 1.05±0.22 #$ |
| **Ti (sec)** | 0.25±0.2 | 0.29±0.12 | 0.17±0.09 | 0.23±0.12 | 0.25±0.24 |
| **Te (sec)** | 0.4±0.18 | 1.44±0.97 * | 0.26±0.14 # | 0.72±0.40 #$ | 0.35±0.2 #@ |
| **FVC (ml)** | 4.99±1.26 | 2.68±1.14 * | 6.07±2.19 # | 3.53±1.56 $ | 6.23±1.99 #@ |
| **FEV1 (ml)** | 3.47±0.82 | 1.22±0.51 * | 4.68±2.05 *# | 2.09±0.96 *$ | 4.49±1.72 #@ |
| **FEV1/FVC**  **(%)** | 70.47±9.27 | 46.16±3.27 * | 75.69±9.34 # | 58.91±7.5 *#$ | 72.28±7.52 #@ |
| **PIF (ml/min)** | 3.22±1.54 | 2.15±0.99 | 2.70±0.91 | 3.30±1.43 | 3.25±1.54 |
| **PEF (ml/min)** | 14.24±5.04 | 6.46±3.65 * | 17.04±8.23 # | 14.31±3.27 # | 15.42±3.46 # |

**Table 5:** Comparison between pulmonary functions parameters in all studied groups *: statistically significant compared to corresponding value in control group (P<0.05) #: statistically significant compared to corresponding value in COPD group (P<0.05) $: statistically significant compared to corresponding value in melatonin group (P<0.05) @: statistically significant compared to corresponding value in MSCs group (P<0.05).

|  | **Control** | **COPD** | **% change** |
| --- | --- | --- | --- |
| **VEGF (ng/mg tissue)** | 3.1±1.56 | 12.48±2.42 * | 302.58 |
| **HIF-1α (FC)** | 1.03±0.13 | 16.09±3.36 * | 1462.14 |

**Table 6:** Effect of COPD on VEGF& HIF-1α expression. Values are presented as mean ±SD. *: statistically significant compared to corresponding value in control group (P<0.05)

|  | **Control** | **COPD** | **Melatonin** | **% change compared to control** | **% change compared to COPD** |
| --- | --- | --- | --- | --- | --- |
| **VEGF (ng/mg tissue)** | 3.1±1.56 | 12.48±2.42 * | 4.27±1.40 # | 37.74 | -65.79 |
| **HIF-1α** | 1.03±0.13 | 16.09±3.36 * | 5.68±1.30 *# | 451.46 | -64.70 |

**Table 7:** Effect of melatonin on VEGF & HIF-1α expression.Values are presented as mean ±SD. *: statistically significant compared to corresponding value in control group (P<0.05) #: statistically significant compared to corresponding value in COPD group (P<0.05)

|  | **Control** | **COPD** | **MSCs** | **% change compared to control** | **% change compared to COPD** |
| --- | --- | --- | --- | --- | --- |
| **VEGF (ng/ mg tissue)** | 3.1±1.56 | 12.48±2.42 * | 7.96±1.40 *# | 156.77 | -36.22 |
| **HIF-1α (FC)** | 1.03±0.13 | 16.09±3.36 * | 8.23±1.28 *# | 699.03 | -48.85 |

**Table 8:** Effect of MSCs treatment on VEGF & HIF-1α expression. Values are presented as mean ±SD *: statistically significant compared to corresponding value in control group (P<0.05) #: statistically significant compared to corresponding value in COPD group (P<0.05)

|  | **Control** | **COPD** | **Melatonin -**  **MSCs** | **% change compared to control** | **% change compared to COPD** |
| --- | --- | --- | --- | --- | --- |
| **VEGF (ng/ mg tissue)** | 3.1±1.56 | 12.48±2.42 * | 3.26±1.37 # | 5.16 | -73.88 |
| **HIF-1α (FC)** | 1.03±0.13 | 16.09±3.36 * | 1.03±0.21 # | 6.89 | -93.6 |

**Table 9:** Effect of combined treatment on VEGF & HIF -1α expression. Values are presented as mean ±SD *: statistically significant compared to corresponding value in control group (P<0.05), #: statistically significant compared to corresponding value in COPD group (P<0.05).

|  | **Control** | **COPD** | **Melatonin** | **MSCs** | **Melatonin - MSCs** |
| --- | --- | --- | --- | --- | --- |
| **VEGF (ng/mg tissue)** | 3.1±1.56 | 12.48±2.42 * | 4.27±1.4 # | 7.96±1.40 *#$ | 3.26±1.37 #@ |
| **HIF -1α** | 1.03±0.13 | 16.09±3.36 * | 5.68±1.3 *# | 8.23±1.28 *#$ | 1.03±0.21 #$@ |

**Table 10:** Comparison between VEGF & HIF -1α in all studied groups. Values are presented as mean ±SD. *: statistically significant compared to corresponding value in control group (P<0.05). #: statistically significant compared to corresponding value in COPD group (P<0.05), $: statistically significant compared to corresponding value in melatonin group (P<0.05), @: statistically significant compared to corresponding value in MSCs group (P<0.05).

| **Group** | **Mean Radial alveolar count ± SD** |
| --- | --- |
| Control | 39.4± 0.69 |
| COPD | 16.2 ± 2.06 * |
| Melatonin | 31.7 ± 0.99 * **^#^** |
| MSCs | 28.1 ± 1.47 * **^# $^** |
| Melatonin - MSCs | 38.9 ± 2.06 **^# $ @^** |

**Table 11:** shows the results of Mean Radial alveolar count ± SD in H&E-stained sections of the studied groups. *: statistically significant vs control group (P<0.05). #: statistically significant vs COPD group (P<0.05). $: statistically vs melatonin group (P<0.05). @: statistically significant vs MSCs group (P<0.05).

| **Group** | **Mean Area % of CD31 ± SD** |
| --- | --- |
| Control | 2.93 ± 0.89 |
| COPD | 11.28 ± 1.41 * |
| Melatonin | 5.18 ± 0.94 * **^#^** |
| MSCs | 7.14 ± 0.63 * **^# $^** |
| Combined | 3.52 ± 0.64 **^# $ @^** |

**Table 12:** shows the results of Mean Area % of CD31 ± SD in immune-stained sections of the studied groups. *: statistically significant vs control group (P<0.05). #: statistically significant vs COPD group (P<0.05). $: statistically vs melatonin group (P<0.05). @: statistically significant vs MSCs group (P<0.05).
